# Maternal Obesity Management Using Mobile Technology: A Feasibility Study to Evaluate a Text Messaging Based Complex Intervention during Pregnancy

**DOI:** 10.1155/2015/814830

**Published:** 2015-04-16

**Authors:** Hora Soltani, Alexandra M. S. Duxbury, Madelynne A. Arden, Andy Dearden, Penny J. Furness, Carolyn Garland

**Affiliations:** ^1^Centre for Health and Social Care Research, Sheffield Hallam University, Collegiate Crescent, Sheffield S10 2BP, UK; ^2^Department of Psychology, Sociology and Politics, Sheffield Hallam University, Collegiate Crescent, Sheffield S10 2BP, UK; ^3^Communication and Computing Research Centre, Sheffield Hallam University, Arundel Street, Sheffield S1 2NU, UK; ^4^Doncaster and Bassetlaw Hospitals NHS Foundation Trust, Armthorpe Road, Doncaster DN2 5LT, UK

## Abstract

*Background*. Maternal obesity and excessive gestational weight gain (GWG) are on the rise with negative impact on pregnancy and birth outcomes. Research into managing GWG using accessible technology is limited. The maternal obesity management using mobile technology (MOMTech) study aimed at evaluating the feasibility of text messaging based complex intervention designed to support obese women (BMI ≥ 30) with healthier lifestyles and limit GWG. *Methods*. Participants received two daily text messages, supported by four appointments with healthy lifestyle midwife, diet and activity goal setting, and self-monitoring diaries. The comparison group were obese mothers who declined to participate but consented for their routinely collected data to be used for comparison. Postnatal interviews and focus groups with participants and the comparison group explored the intervention's acceptability and suggested improvements. *Results*. Fourteen women completed the study which did not allow statistical analyses. However, participants had lower mean GWG than the comparison group (6.65 kg versus 9.74 kg) and few (28% versus 50%) exceeded the Institute of Medicine's upper limit of 9 kg GWG for obese women. *Conclusions*. MOMTech was feasible within clinical setting and acceptable intervention to support women to limit GWG. Before further trials, slight modifications are planned to recruitment, text messages, and the logistics of consultation visits.

## 1. Background

Obesity (body mass index (BMI) ≥ 30 kg/m^2^) is a major public health challenge, affecting 25% of the UK adult population [1].

Women of childbearing age are similarly at risk of obesity with currently approximately 20% of pregnant women being obese, imposing growing demands on health service provision [2]. Obesity is associated with a significant rise in the risk of maternal and neonatal mortality and morbidity [3]. The adverse outcomes of obesity and excessive weight gain during pregnancy may include gestational diabetes, preeclampsia, postpartum haemorrhage, urinary and genital tract infection, wound infection, caesarean section, induction of labour, and instrumental birth as well as poorer neonatal outcomes such as congenital anomalies [4], macrosomia, and fetal death [3, 5]. In addition, maternal obesity and excessive gestational weight gain are associated with further development of central adiposity in mothers [6] and an increased risk of obesity in the offspring [7]. Exploring new interventions to address obesity in pregnancy could lead to improving child and maternal health and to save NHS resources.

Although behavioural and pharmacological interventions for obesity in the general population are well explored, UK based research into the efficacy of weight management interventions in pregnancy is limited [8]. Up to 60% of obese women and 68% of overweight women gain excessive amounts of weight during pregnancy [9]. A number of international studies [10–14] and systematic reviews [15, 16] have evaluated interventions designed to control weight gain in pregnancy with various results. Thangaratinam et al. [16] reported a meta-analysis of 44 randomized control trials looking at the effects of interventions in pregnancy on maternal weight and obstetric outcomes including diet only, exercise only, and mixed methods trials and found that some diet, exercise, and mixed interventions led to a reduction in gestational weight gain and improved obstetric outcomes but dietary interventions were the most effective. However due to the inconsistencies in the quality of the included studies more researches—particularly UK based studies—are required in this area [8].

None of the above trials used text messaging as a support mechanism to deliver a mixed intervention including diet and physical activity to promote healthy gestational weight gain. This is important as other investigators [17] have suggested that future research should focus upon novel and creative modes of intervention such as telephone and Internet based programmes. Mobile technology is portable, instantly accessible, and private and can be individually tailored [18, 19] meeting health policy aspirations for personalization in health care delivery. Text messaging in particular may be more cost-effective than traditional weight management interventions [20] which typically involve individual or group sessions delivered by health professionals. A wide public access to mobile phones makes it a worthwhile option to be incorporated into interventions for behaviour change purposes.

Several studies have reported successful results in weight loss interventions using text messaging [18, 21–25] in the general public. Similarly Hurling et al. [26] reported significant weight loss and increased physical activity from their Internet and text messaging based interventions. Except for a small pilot study which has recently reported the practicality of the use of text messaging in gestational weight management trials [27], this has not yet been evaluated in pregnant women.

We have therefore developed the MOMTech project involving a complex intervention with several interconnecting compartments [28] to address maternal obesity management during pregnancy. MOMTech includes a text messaging based complex intervention integrating evidence based health behaviour change techniques in support of weight management. These were derived from evidence suggesting that across both healthy eating and physical activity interventions the most effective interventions were those which combined self-monitoring [29–31] with at least one other technique from control theory [32, 33] such as setting goals, receiving feedback, and reviewing goals in light of feedback. In our intervention, text messaging was used to facilitate a wide platform for delivering such health behaviour change strategies. This feasibility study was designed to evaluate the practicality of the above intervention prior to a large well designed multicentre trial, drawing on evidence based behaviour change strategies and commonly used communication technologies.

## 2. Aims and Objectives

This feasibility study aimed to explore the appropriateness of a text messaging based complex intervention for promoting healthy gestational weight gain during pregnancy.

The main objectives were

- to assess the acceptability of text messaging and compliance with the intervention,
- to assess the acceptability and feasibility of completing the diet and activity record,
- to assess the practical issues for clinicians operating the technology as part of their clinical consultations,
- to identify areas for improvement to refine the intervention accordingly,
- to assess recruitment process and attrition pattern.

## 3. Methods

### 3.1. Design

A mixed methods approach comprising qualitative and quantitative components was used. The quantitative components included a single arm intervention featuring text messages to improve diet and lifestyle during pregnancy. All women who declined to participate or who had initially verbally agreed and then changed their minds were included in the comparison group for their routinely collected data. The qualitative component included a focus group and interviews with participants, the midwife who delivered the intervention, and those who declined receiving the intervention.

### 3.2. Data Analysis

Descriptive statistics are presented for the quantitative data and a thematic analysis was used for the qualitative data [34].

### 3.3. Setting

The study was conducted in Doncaster in the North East of England where a maternal obesity service for women known to users as “Monday Clinic” has been running for several years. This service is for pregnant women with BMI of 40 and over in which a healthy lifestyles midwife provides diet and lifestyle advice and support over three antenatal appointments at 16, 28, and 36 weeks at this clinic. Through previous local research and service evaluation, staff and maternity users identified a need for a supportive service such as text messaging to build on the existing clinic [35, 36] without increasing clinic contact time.

### 3.4. Ethics

Ethical approval was granted from the National Research Ethics Service Committee Yorkshire and Humber-South Yorkshire. Women were informed that participation was voluntary, that they could withdraw at any time, and that all data will be confidential. Women who declined or withdrew from the study consented for their routine data to be used for analysis and this was recorded in their antenatal records.

### 3.5. Recruitment

Pregnant women with a BMI ≥30 and aged 18 years and over who were accessing maternity units in Doncaster Royal Infirmary Hospital were invited to participate in the booking visit (8–10 weeks) by five community midwives between end of July 2013 and early January 2014. Due to the nature of the intervention, participants had to be able to read and understand English language. They were excluded if they had a history of complications such as diabetes, hypertension, antepartum haemorrhage, unexplained fetal loss/stillbirth, psychiatric illness, or a multiple pregnancy.

Participants received a £10 gift voucher on completion of each of the three questionnaires at 18, 28, and 36 weeks, and those who took part in the focus groups and interviews were reimbursed for their time and travel.

### 3.6. Intervention Protocol

The details of intervention protocol are summarized in Table 1. After referral at the booking visit, the intervention was delivered by the specialist healthy lifestyle midwife and included predesigned text messages as well as four antenatal visits at the hospital (two consultation visits and two follow-up clinic appointments). This also included a self-monitoring element consisting of a one-week food and activity diary before consultation 2 and a diet and activity record (DAR) completed after consultation 2 to reflect on progress towards goals. To reduce the size of the DAR, this was split to two booklets and was given to women at two contact points (16–18 and 28 weeks).

Consultation 1 provided an opportunity for women to familiarize themselves with the study, to start a one-week food and activity diary, and to select a message from a series of motivational text messages which they received at specified times of the day, before the next consultation. At this stage women received one message per day.

At consultation 2, women were encouraged to reflect on their dietary and physical activity behaviour and to select one of five healthy eating goals and one of three activity goals. The five healthy eating goals were “I am not going to overeat in pregnancy,” “I am going to eat five portions of fruit and vegetables every day,” “I am going to eat healthy snacks and drink low-calorie drinks,” “I am going to eat three balanced meals every day,” and “I am going to make healthy family food choices.” The physical activity goals were “I am going to walk and take the stairs whenever I can,” “I am going to exercise for at least 10 minutes every day,” and “I am going to exercise for at least 30 minutes every day.” Women selected text messages associated with their chosen goals and the time when these messages would be delivered. These goals were recorded in their DARs, which they completed weekly from consultation 2 onwards and were implicitly prompted to be completed by the text messages.

After consultation 2, the text messages were delivered twice daily (one in line with the mothers' physical activity and another one aligned with their dietary goals) and received at a preselected time by mothers. They were also one way; however, women could send a “STOP” message to opt out at any time they wished to do so. The messages in consultation 1 were broadly motivational allowing the participants to become accustomed to the intervention and to feel positive that their behaviour could have a positive effect on their health and the health of the baby. The text messages included information about health consequences and persuasion about capability [37], for example,* “Every day that you eat healthily and exercise makes a difference to your baby: You can do it!”*

The messages in consultation 2 were grouped according to each of the healthy eating and activity goals. For each goal there were four categories of messages:

*(1) Motivation*. For example,* “Many women make healthy changes during pregnancy for themselves and their growing baby. Be one of them!”*

*(2) Specific Planning (Action Planning)*. For example,* “Plan exactly when and where you are going to do 10 minutes of exercise today.”*

*(3) Overcoming Barriers (Coping Planning)*. For example,* “If you are feeling tired, then remember that exercise in the daytime will help you to sleep at night.”*

*(4) Self-Monitoring*. For example,* “Take a look back at your Diet and Activity Record. How have you done this week? Well done on making the effort and think about how you can make even healthier choices next week.”*

There were between 11 and 16 text messages for each of the eight goals, with an initial total of 103 standardized messages. Some of the standard messages were editable to be personalized, for example, “Take (NAME of other child) for a walk in the park today: good for you and them.”

The two follow-up appointments were combined with routine antenatal appointments at 28 and 36 weeks to review progress in the diet and activity records (DARs), weigh participants with feedback, and change text messages or goals if required. Additional data were collected for the study by researchers who were not involved in delivering any part of the intervention, as summarized in Table 2.

## 4. Results 

### 4.1. Recruitment and Attrition Rates

A total of 28 women verbally agreed and six women declined to take part in the study with this initial decision recorded by a sticker on their notes completed by their community midwife. We emphasized to the five designated community midwives the importance of recording the total number of women invited to the study; however the use of the sticker system was inconsistent at times. From the recorded number of women who were approached from the sticker system, an uptake of 47% (16/34) was observed.

**Figure 1:**
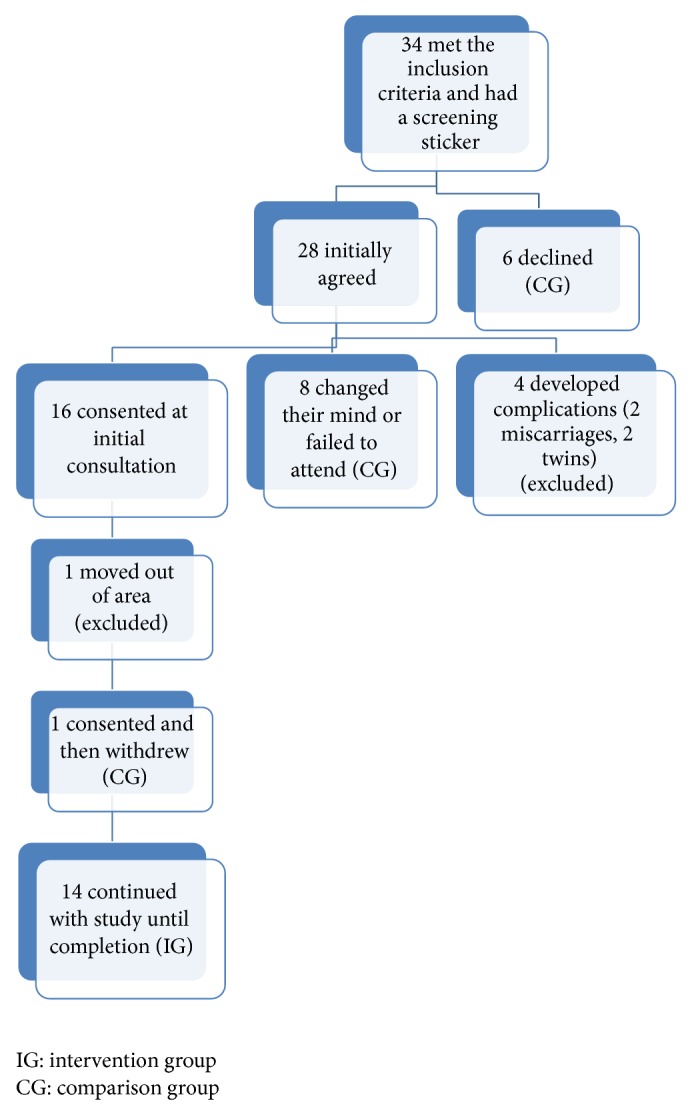
Participant recruitment summary.

Of 28 women who verbally agreed, 16 women provided informed consent at their first appointment with the specialist midwife, although one later moved out of area and one did not attend any further visits, leaving a cohort of 14 women who all successfully completed the study from 14 weeks until delivery. The remaining 12 out of the 28, who originally verbally agreed to participate but did not provide written consent, consisted of eight (28%) who changed their minds and four (14%) who were excluded due to medical/clinical complications. For data analysis purposes we have compared the 14 participating women (intervention group (IG)) with those originally declined (6), those who changed their mind before written consent (8), and the one who did not attend after consenting (1), making a comparison group (CG) of 15 in total (see Figure 1).

### 4.2. Women's Characteristics

The baseline demographics for participants and women who declined to take part in the study are presented in Table 3. Due to small number of participants and those available for comparison no statistical analyses were applied. The groups appear to be similar in all demographic characteristics except for parity where a higher proportion of intervention group was nulliparous (35.7% versus 6.7% in the comparison group). It also appears that the intervention group has a higher baseline weight than the comparison group but their BMI values seem to be similar.

### 4.3. Maternal and Infant Outcomes

From a visual inspection of Table 4, women in the intervention group seem to have a considerably lower amount of gestational weight gain (mean (SD) kg 5.6 (4.6) versus 9.7 (7.2)) in relation to the comparison group. They were also less likely to exceed the Institute of Medicine (IOM) [11] gestational weight gain limit of 9 kg (28% versus 50%, resp.). There were no incidences of large for gestational age or small for gestational age in the study groups. One stillbirth was reported in the comparison group, being born at 34 gestational weeks.

### 4.4. Intervention Process

The goal setting, text messaging, and self-monitoring tools only applied to women who participated in the intervention. These were aimed to support behaviour change as part of the complex intervention.

### 4.5. Goal Setting and Text Message Selection

Out of the eight predefined goals, the most popular health eating goal was “I am going to eat three balanced meals every day” and the most commonly selected physical activity goal was “I am going to exercise at least 30 minutes every day.” None of the participants selected the goal entitled “I am going to eat five portions of fruit and vegetables every day” (see Table 5).

Along with selecting the standard messages relating to their goals, women had the option of choosing editable messages which appeared to be highly popular. The healthy lifestyle midwife adapted these editable messages creating 63 additional messages which included praising statements. The examples for this include* “Heather, keep on trying to be active and do whatever you can—I'm proud of you for trying!”*

### 4.6. Self-Monitoring Tools

#### 4.6.1. One-Week Food and Activity Diary

Every participant completed the initial 1-week food and activity diary, although two participants forgot to complete it in time for consultation 2, so they brought it to their next appointment.

#### 4.6.2. Diet and Activity Records

Half the participants returned two completed DARs. Ten of the first 16–28 week DARs were returned and eight of the 29–36 week DARs were returned.

### 4.7. Qualitative Findings

A qualitative phase was conducted to further explore the practical aspects of MOMTech and experiences of the specialist midwife and participants as well as reasons for lack of participation for those who declined or withdrew from the study and these thematic findings are presented below.

### 4.8. Comparison Group

#### 4.8.1. Reasons for Lack of Participation

A convenience sample of eight women from the comparison group was contacted, of whom four women agreed to a short telephone interview.

Reasons given for declining to take part in MOMTech are clustered into the following four themes: recruitment/approaching style, timing of the intervention, low perception of risk, and the fact that some decliners thought that MOMTech would not benefit them but might benefit first time mums.

*Recruitment and approaching style* included women feeling judged and treated differently because of their size. They felt that the recruiting midwife only mentioned it because of their increased BMI, not fully understanding why it was limited to obese women.

*Timing of the intervention* covered beliefs that pregnancy was not a good time as you are expected to gain weight and develop physical health problems and that there was a lack of suitable antenatal exercise classes in the area even if you wanted to be active. They also stated issues related to balancing participation with the demands of work and their existing family and that this intervention would be more beneficial to first time mums.

*Low perception of risk* comprised not being fully aware of the impact of a high BMI or excessive weight gain on pregnancy. Some women did not see their weight as a health issue as they felt they were fat and healthy, not that big anyway, or that obesity is normal; others were very sensitive and self-conscious about their weight, so they did not want it to be regularly discussed and to be weighed. There was confusion over the additional risks due to obesity and weight gain, as they reported having slim friends who had gained excessive weight, so they did not understand why we exclusively recruited obese women.

*The fact that some decliners thought that MOMTech would not benefit them but might benefit first time mums* included the perception that they had learnt the hard way from their previous pregnancies, so they did not need support to be healthy and if they wanted to be healthy they could do it themselves. However they acknowledged that first time mums might find it informative and motivating with the texts useful to keep them on track and prevent them from making the same mistakes they made.

### 4.9. Intervention: Participation and Delivery

#### 4.9.1. Experiences of Participants

A convenience sample of women from the intervention group was contacted approximately six weeks after delivery and invited to participate in a focus group or telephone interview to explore experiences of participating in the study and how the intervention could be improved. A focus group was conducted with two participants (four invited and two attended) and telephone interviews were done with three participants a few weeks apart due to the staggered recruitment.

Their feedback on the intervention is presented in the following four main themes: personalized support and praise, healthy habit formation, the fact that they thought it was keeping them on track, and intervention design, tools, and resources.

*Personalized support and praise* consisted of many comments about the ongoing support via regular contacts and the positive nonjudgmental approach used throughout the intervention. They liked the holistic nature of the intervention and its focus on mum's health rather than focusing purely on the baby's as a “mother is more than just a baby carrier,” having perceived routine care provided in previous pregnancies to be predominantly focused on the baby.

*Healthy habit formation* included finding the text messages, 1-week food and activity diary and DARs useful in identifying areas to change, reflecting on progress towards their goals, and reinforcing the behaviour changes; for example, for one woman a message at 11 am every day was a reminder to eat a healthy snack, or for another a regular message about walking prompted them to go for a walk even on a dull day. Some suggested we should do a postnatal phase of regular text messaging to continue healthy behaviours and to support breastfeeding, weight loss, and good mental health.

*The fact that they thought it was keeping them on track* included feeling motivated to try and stick to their goals, even when external factors such as work pressure or pelvic pain was making it more challenging to do so. A few mentioned feeling like someone was on their shoulder watching them to make sure they made healthy choices. They all liked being weighed and receiving praise from the midwife for their efforts as they could feel that the intervention was working for them.

*Intervention design, tools, and resources *included agreeing that the number of appointments was ideal, that monthly phone calls were unnecessary, and that in the future the intervention could be delivered by specialist or community midwives with training. The initial food and drink diary was beneficial as it highlighted eating patterns in general, with the DAR being useful as it was quick to complete weekly. The texts were well received but some participants requested the option to change them at monthly intervals if they were finding them less useful or their circumstances changed. The monthly phone calls from the midwife to allow change of the text messages were part of the protocol but this did not take place. Personalized messages were considered the most useful and having to select from the four categories was considered by some to be a bit challenging/restrictive. Many comments focused around personalization, either of the messages and goals or of the intervention itself, with future participants having the choice to do some or all of the elements of the intervention, perhaps with additional support from more 1-week food diaries or a pedometer when they felt they needed it.

#### 4.9.2. Experience and Suggestions from the Specialist Midwife

Feedback from the specialist midwife is presented in the following three themes: relationship building, personalized support, and information technology (IT) and logistics.

*Relationship building* was a major advantage of the intervention allowing the midwife to fully understand the woman's lifestyle and build a trusting relationship over the appointments. This resulted in a genuine care and interest in the women's pregnancy journey sharing not only the excitement of appropriate weight gain but also some of the responsibility if the healthy behaviour change was not as expected.

*Personalized support* was seen as a benefit from the midwife and the women. She was able to draft personalized messages praising their efforts and tailor them to their circumstances which made the midwife feel good that she could support women in trying to achieve a healthy lifestyle behaviour change. She was also able to draw on information about their family and home life to enable them to support the woman and could use her position and contact women to refer them to additional services as required making it a holistic intervention rather than just focusing on weight or healthy eating.

*Information technology and logistics* included ways in which the face to face sessions could be better organized to utilize the time efficiently by streamlining consultation 2 and having a shorter list of messages to select from and simplifying few stages on the IT system to be able to quickly select, save, and print the messages.

## 5. Discussion

This provisional study showed that a text messaging based intervention for limiting gestational weight gain is feasible in terms of application in a clinical setting and acceptable by obese pregnant women. The recruitment took approximately six months with 16 eligible women providing written consent at the study onset and an attrition rate of 2/16 (12.5%). The longer than expected recruitment period could be due to its coincidence with the school summer holidays as well as usual issues with a slow start and engagement processes at the beginning of any study.

Although ultimately we are aiming to evaluate the intervention in terms of its impact on healthy lifestyle behaviours and on pregnancy and birth outcomes such as birth weight and gestational weight gain, the small sample size of this feasibility study was not statistically powered to detect these changes. Nevertheless, a trend towards reduction in gestational weight gain was observed in women who participated in the intervention compared to those who declined participation. This positive trend towards a healthier birth outcome could be due to the selection bias and that women who participated in the study may have been more motivated in adapting a healthy lifestyle than the comparison group. A comparative statistical analysis was not deemed appropriate from a visual inspection; however, the baseline characteristics of women who participated and those who did not seem fairly similar except for parity, where the number of multiparous women who declined the study was greater than the number of those who participated.

To our knowledge, there is only one other study [27] which has piloted the use of text messaging in pregnancy and similarly showed positive evidence for practicality of such technology as an intervention to reduce gestational weight gain. There were differences in their methodology as it was a pilot randomized trial comparing tailored two-way text messaging related to personal goals in key areas versus generic text messages but without the additional clinic support. They were also focusing on overweight and obese, whereas our study population included only obese women. This is important as literature suggests a different pattern of response to gestational weight management interventions according to BMI categories [16, 38].

It is worth highlighting that LIMIT [14], the recent large randomized control intervention in Australia which primarily compared a comprehensive lifestyle intervention to standard care to reduce the incidence of large for gestational age (LGA), did not show a significant reduction in LGA, in average gestational weight gain, or in proportions of participants exceeding the IOM guidelines. However, they did show a significant reduction in the incidence of macrosomia (birthweight > 4 kg) in the intervention compared to the control group. These results related to a total of 2212 overweight and obese women collectively and no subgroup analysis based on BMI category was reported. The lifestyle intervention included some of the elements we have used such as providing advice on diet and physical activity, identifying barriers and problem solving, setting goals, and self-monitoring in a workbook; however it was delivered via three face to face meetings and three telephone calls by a research dietician and research assistants rather than mainly at the time of routine visits (except for the consultation 2 visit) and via text messaging in our intervention.

Like Pollak et al. [27], our intervention has the advantage over all other existing studies in this area in utilizing a text messaging service for a continuous support between contacts and health professionals to maintain engagement and encourage positive behaviour change. Our qualitative data show participants' appreciation of having a consistent reminder for habit formation to keep them on track between visits.

In general, the intervention was delivered as defined in the study protocol. The minor deviations included the abandonment of the monthly phone calls due to additional time demand for the midwife and inconvenience for the women. Although the recruitment was satisfactory, we were unable to calculate an accurate uptake rate from our sticker system. In the future trial, this could be overcome by a more regular communication and training for community midwives during the recruitment period.

Text messages were generally well received; however several areas including message selection and personalization were identified for future improvement through the qualitative element of the study. Some participants indicated that they would like to change their messages during pregnancy and after the monthly phone calls were abandoned; they were only offered this at the 28- and 36-week appointments; however very few actually did change their messages. One solution could be to develop the system to accept a message such as “CHANGE” so women could text the platform back and the midwife could then call and arrange to update the messages with those participants. We had 103 messages; however women were only exposed to the set of messages relating to their goals. The future modifications may include having a set list of automated motivational and self-monitoring messages but still allow women to choose their overcoming barriers and specific planning messages from a shorter list of options. Women received 14 messages a week but this could be reduced to make them receive six to ten messages weekly to simplify the selection process. The participants seemed to particularly like the personalized messages which offered positive reinforcement and praise. The specialist midwife created 62 unique personalized simple messages which were slightly different from the predesigned editable text messages including praising statements. Our system will allow these simple rewarding messages to be standardized and yet be editable with details such as the name of the woman or relatives to be incorporated in the future developments. This minimizes the burden for the person who delivers the intervention and maintains the element of personalization as well as consistency.

The high retention rate and satisfactory completion of study tools including the food and drink diary, DARs, and questionnaires demonstrate that once the women were recruited and consented at consultation 1, they were very engaged and motivated to participate. This was also captured in the qualitative data with participants feeling they benefitted from the intervention, would do it again on subsequent pregnancies, and would recommend it to other women and they had stated that they continued with some of the behaviours into the postnatal period. This is in line with the findings from only existing pilot study evaluating the feasibility of text messaging in pregnancy [27].

In terms of staff time and delivering the intervention, there were a few areas suggested for improvement: to make the intervention more effective, to reduce the time demand on staff, and to facilitate replication in other settings. These will be addressed in the modification of the intervention by standardizing the editable messages, shortening the list of text messages, and providing intensive training when implementing the full trial.

## 6. Conclusions 

This study indicates that using a text messaging based complex intervention for managing gestational weight gain is feasible in a clinical setting and is acceptable by women. Before trialling in other settings, slight modifications to the recruitment process, text messages, and the logistics of consultation visits will be made.

## Figures and Tables

**Table 1:** Intervention protocol summary.

Booking visit 10 weeks	Collect height and weight measurements to check BMI is ≥30
	Explain study, give information leaflet, and record verbal interest with sticker on notes

Consultation 1 14–16 weeks	Confirm eligibility, explain study, obtain written consent, and build rapport with the woman
	Enter mobile number and due date into the system
	Explain the initial goal setting and text messages, selecting days and times
	Measure woman's weight and height measurements to confirm BMI and share with her
	Give 1-week food and activity diary to complete by next visit

Consultation 2 16–18 weeks	Review the food and activity diary and use this information to guide woman to select two goals
	Agree on one diet and one physical activity goal
	Select 6–10 text messages from a predefined list relevant to those goals and specify times and days
	Explain the diet and activity record and how to complete it weekly
	Measure woman's weight and share with her

Follow-up appointment 28 weeks	Review the diet and activity record, current goals, and texts and change texts/goals as required
	Collect first diet and activity record (16–28 weeks); issue the second one (29–36 weeks)
	Measure woman's weight and share with her

Follow-up appointment 36 weeks	Collect diet and activity record, and reflect on progress
	Measure woman's weight and share with her

Telephone calls at 20, 24, and 32 weeks	Review progress, set new goals, or change texts as required

**Table 2:** Data collection schedule.

Consultation 1	Record participant baseline characteristics and previous maternal history

Between consultations 1 and 2	Complete the food frequency questionnaire (FFQ), pregnancy physical activity questionnaire (PPAQ), and psychosocial questionnaire, EQ5D-3

28 weeks	Complete the FFQ, PPAQ, and psychosocial questionnaire, EQ5D-3

36 weeks	Complete the FFQ, PPAQ, and psychosocial questionnaire, EQ5D-3

After delivery	Examine routine medical notes and record pregnancy, delivery, maternal, and infant outcomes

6 weeks postpartum	Conduct focus groups or interviews by researcher with participants, controls, and the specialist midwife who delivered the intervention

**Table 3:** Women's characteristics in the intervention and comparison groups.

Characteristic	Intervention group *n* = 14	Comparison group *n* = 15
	Mean (SD) [Range]	Mean (SD) [Range]
Age (years)	29.1 (5.4) [22.0–38.0]	31.7 (5.8) [22.0–43.0]
Height (m)	1.6 (0.7) [1.5–1.8]	1.7 (0.7) [1.5–1.8]
Booking weight kg	99.4 (14.6) [75.0–131.6]	107.6 (13.2) [96.0–131.0]
Booking BMI	36.6 (4.5) [31.1–45.0]	37.0 (5.4) [31.0–46.8]

	*n* (%)	*n* (%)

Parity		
Nulliparous	5 (35.7)	1 (6.7)
Multiparous	9 (64.3)	14 (93.3)
[Range]	[0–3]	[0–4]
White British	14 (100.0)	15 (100.0)
Marital status		
Married	6 (43.3)	5 (33.3)
Cohabiting	7 (50.0)	9 (60.0)
Single	1 (7.0)	2 (13.3)
Employment	11 (78.0)	9 (60.0)
Smokers	4(28.6)	5 (33.3)

**Table 4:** Maternal and neonatal outcomes in the intervention and comparison groups.

	Intervention group *n* = 14	Comparison group *n* = 14^*^
	Mean (SD) [Range]	Mean (SD) [Range]
Gestational weight gain (kg)	5.6 (4.6) [0.0–15.0]	9.7 (7.2) [−2.0–21.2]^&^
Birthweight (gr)	3598.7 (532.8) [2785.0–4390.0]	3453.2 (525.1) [2120.0^¥^–4200.0]
Gestational age	39.3 (1.5) [36.0–41.0]	39.2 (1.8) [34.0^¥^–41.0]

	*n* (%)	*n* (%)

Mode of birth		
Spontaneous vaginal birth	8 (57.1)	8 (57.1)
Caesarean section	6 (42.9)	4 (28.6)
Instrumental birth	0	2 (14.3)
Induction of labour	5 (5.7)	3 (21.4)
Gender		
Female	11 (78.6)	12 (85.7)
Male	3 (21.4)	2 (14.3)
Large for gestational age	0	0
Small for gestational age	0	0
Stillbirth	0	1
Gestational weight gain (GWG) in relation to IOM guidelines		
GWG < 5 kg	7 (50.0)	4 (33.3)^&^
GWG 5 kg to 9 kg	3 (21.4)	2 (16.7)
GWG > 9 kg	4 (28.6)	6 (50.0)

^*^Although there were originally 15 in the comparison group, one moved away so we could not access their maternal and infant outcome data.

^&^Data was only available on 12 women for gestational weight gain outcome as one gave birth at the 34th week and data on other women was not reported.

^¥^The lowest value relates to the only stillborn baby in the cohort.

**Table 5:** Frequency of selected goals.

Healthy eating goals	*n*
I am going to make healthy family food choices	1
I am not going to overeat in pregnancy	2
I am going to eat five portions of fruit and vegetables every day	0
I am going to eat healthy snacks and drink low-calorie drinks	4
I am going to eat three balanced meals every day	7

Physical activity goals	*n*

I am going to walk and take stairs whenever I can	3
I am going to exercise at least 10 minutes every day	4
I am going to exercise at least 30 minutes every day	7

## List of Abbreviations

GWG: Gestational weight gain
IOM: Institute of Medicine
BMI: Body mass index.
